# Nanotechnology, bionanotechnology and microbial cell factories

**DOI:** 10.1186/1475-2859-9-53

**Published:** 2010-07-05

**Authors:** Antonio Villaverde

**Affiliations:** 1 Institute for Biotechnology and Biomedicine, Universitat Autònoma de Barcelona, Bellaterra, 08193 Barcelona, Spain; 2 Department of Genetics and Microbiology, Universitat Autònoma de Barcelona, Bellaterra, 08193 Barcelona, Spain; 3 CIBER de Bioingeniería, Biomateriales y Nanomedicina (CIBER-BBN), Bellaterra, 08193 Barcelona, Spain

## Abstract

Nanotechnology is increasingly using both materials and nano-objects synthesized by living beings, most of them produced by microbial cells. Emerging technologies and highly integrative approaches (such as 'omics and systems biology), that have been largely proven successful for the production of proteins and secondary metabolites are now expected to become fully adapted for the improved biological production of nanostructured materials with tailored properties. The so far underestimated potential of microbial cell factories in nanotechnology and nanomedicine is expected to emerge, in the next years, in the context of novel needs envisaged in the nanoscience universe. This should prompt a careful revisiting of the microbial cell factories as the most versatile biological platforms to supply functional materials for nanotechnological applications.

## 

Generally speaking, Nanotechnology refers to the fabrication, manipulation and utilization of submicron objects, particularly those between 1 and 100 nm. Physical and chemical sciences have developed tools and procedures to fabricate nanoscale entities with intriguing applications in electronics, material sciences and medicine. In the biomedical context, the relevance of Nanotechnology relies on the particular biophysical properties of nanoscale objects and their particular interaction with living beings such as high diffusion in organs and tissues, high surface-volume ratio, efficient uptake by mammalian cells and high biological impact in biological interfaces through mecano-transduction signaling [1] and a spectrum of alternative cell activities and responses [2]. The extraordinary bio-effectiveness of nanoparticles has in turn derived into a strong debate about their potential toxicity, when directly exposed to the human body or released to the environment [3], which is still unsolved. The biological impact of these manmade nanoscale entities and their suitability to be functionalized for specific binding or to act as carriers for therapeutics empowers a spectrum of specific applications in diagnosis and therapy, including imaging, biosensing, regenerative medicine, drug delivery and gene therapy. The clinically-oriented fabrication, tailoring and application of bio-active nanoparticles conceptually sustains the Nanomedicine framework.

Bionanotechnology (as well as Nanobiotechnolgy) are rather fuzzy terms whose overlapping meanings are under continuous refining, as their associated technologies and applications keep evolving. They are often understood as the generation of hybrid materials (deriving from chemical and biological synthesis), or bio-inspired materials [4]. In a different reading frame, Bionanotechnology can be observed as "Nanotechnology through Biotechnology" [5], that is, the bio-fabrication of nano-objects, or bi-functional macromolecules usable as tools to construct or manipulate nano-objects. Because of their wide physiological diversity, small size, genetic manipulability and controlled culturability, microbial cells are ideal producers of a diversity of nanostructures, materials and instruments for Nanosciences, ranging from fully natural products such as viruses, polymers and magnetosomes, to engineered proteins or protein constructs such as virus-like particles (VLPs), and peptide-displaying phages or cells and tailored metal particles (Figure 1).

**Figure 1:**
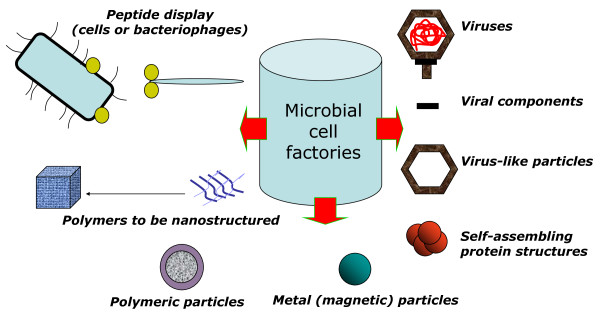
**Biosynthetic potential of Microbial Cell Factories in Nanosciences**. Bacteria and other microbes are good producers of particulate entities with values in Nanotechnology in general and in Nanomedicine in particular. **From top left, clockwise**: First, cells themselves and their infecting viruses are used for peptide display (cell surface display or phage display technologies, respectively). Among other applications including selecting ligands for receptor mediated drug delivery, biosensing or imaging [27-31], peptide display show important promises in molecular biomimetics, to generate molecular links between synthetic and biological components of hybrid materials [32,33]. Also, animal, plant and bacterial viruses, being manageable at the nanoscale, are used as scaffolds for nanofabrication of electronic components [34] and as building blocks for functionalized surfaces [35,36], apart from their more conventional application as vehicles for the delivery of nucleic acids in gene therapy [37,38]. Interestingly, viral components as the DNA-packaging motor of phi29 bacteriophage [39] have been explored as vehicles in drug delivery. VLPs, produced in both eukaryotic microbes and in bacteria, apart from their conventional application in vaccination show promising potential as nano-containers for drug delivery [40]. Other protein self assembling complexes produced in bacteria such as flagella, explored to generate nanomotors or as templates for nanofabrication [41-43], or inclusion bodies, used as soft-matter scaffolds in tissue engineering [44-46] or as functional materials [47] are under deep exploration and further development. Magnetosomes from magnetotactic bacterial have shown exciting potentials in drug delivery, imaging and tissue engineering [48-53], while a diversity of metal nanoparticles produced in bacteria, whose properties can be tuned during production, are in use or under development for nano-electronics, therapy and imaging [54-60]. Main microbial polymers including polysaccharides, polyesters and polyamides can be nanostructured upon isolation from producing cells for uses in material sciences, while others, such as gellan, dextran, PHA and HA, are naturally produced as nanoparticulate materials [61-65], than can be further functionalized in vivo by the genetic engineering of producing cells [66,67].

In summary, microbial cells are natural producers of (or they can be easily adapted to synthesize) nano-sized entities with relevant biomedical applications, being the cell factories themselves promising tools for the emerging technologies and biomedical applications related with the use of nanosized entities. Microbial Call Factories, the journal, has addressed some of these relevant topics through primary research papers and reviews. For instance, Chen and coauthors [6] have recently shown how polyhydroxyalkanoate (PHA) nanosized granules are convenient instruments for protein purification, while other authors [7] have improved the production of clinically relevant microbial materials suitable for nanoparticle in vitro fabrication including alginate, hyaluronic acid (HA) and PHA [8,9].

Moreover, cell surface display technologies [10,11] as well as the engineering of bacterial particulate materials (such as spores) for peptide display [12] have been represented in our article list. Furthermore, the journal has often addressed protein quality issues [13-19], that are highly relevant to the design and production of protein complexes and protein nanoparticles, among which virus like particles (VLPs) [20], self-assembling silk-like proteins [21] and bacterial inclusion bodies [22-26] have been particularly studied.

However, the number of submissions dealing with Nanotechnological applications or instruments deriving from microbial cells and the spectrum of coverage of Nanoscience-related topics are still low. Therefore, the editorial board of Microbial Cell Factories is pleased to encourage all the authors working in microbiology, biomedicine and biomaterial sciences to consider the potential of the Cell Factory platforms and to submit their primary research data and reviews to the journal. As a fully settled but highly dynamic journal, Microbial Cell Factories is committed to fully cover emerging technologies and scientific areas of hot interest from which microbial products are relevant, provided the biological aspects of the production (the Cell Factory concept) of the model particles, structures or materials are conveniently stressed.
